# Age-dependent regenerative mechanisms in the brain

**DOI:** 10.1042/BST20230547

**Published:** 2024-11-25

**Authors:** Giada Vanacore, Jens Bager Christensen, N. Sumru Bayin

**Affiliations:** 1 Gurdon Institute, University of Cambridge, Cambridge, U.K.; 2 Department of Physiology, Development and Neuroscience, University of Cambridge, Cambridge, U.K.

**Keywords:** adaptive reprogramming, neural stem cells, regeneration, rodent brain

## Abstract

Repairing the adult mammalian brain represents one of the greatest clinical challenges in medicine. Injury to the adult brain often results in substantial loss of neural tissue and permanent functional impairment. In contrast with the adult, during development, the mammalian brain exhibits a remarkable capacity to replace lost cells. A plethora of cell-intrinsic and extrinsic factors regulate the age-dependent loss of regenerative potential in the brain. As the developmental window closes, neural stem cells undergo epigenetic changes, limiting their proliferation and differentiation capacities, whereas, changes in the brain microenvironment pose additional challenges opposing regeneration, including inflammation and gliosis. Therefore, studying the regenerative mechanisms during development and identifying what impairs them with age may provide key insights into how to stimulate regeneration in the brain. Here, we will discuss how the mammalian brain engages regenerative mechanisms upon injury or neuron loss. Moreover, we will describe the age-dependent changes that affect these processes. We will conclude by discussing potential therapeutic approaches to overcome the age-dependent regenerative decline and stimulate regeneration.

## Introduction

Regeneration is the process of replacing missing or damaged cells and structures to restore the integrity of the tissue and its function. However, regenerative capacity varies greatly across species, tissues and stages. In contrast with fish and salamanders, which can regenerate their nervous systems throughout life [1,2], the mammalian brain shows a dramatic decline in its regenerative capacity after development, eventually leading to limited repair in the adult brain. The molecular and cellular mechanisms that facilitate or inhibit regeneration in the mammalian brain are poorly understood. This significant gap in our knowledge impairs the design of effective therapeutic strategies that stimulate and promote regeneration in the brain.

The mammalian brain was long considered incapable of generating new neurons after development, a limitation described by Ramon Cajal as a ‘*harsh decree*’ [3]. However, the identification of adult neurogenesis in specific regions of the brain, termed the canonical neurogenic niches, has broken this dogma [4,5]. Despite the presence of neural stem cells (NSCs), the adult brain exhibits a limited capacity to regenerate upon cell loss due to injury or degeneration, outside of the canonical neurogenic niches and adult neurogenesis. Interestingly, during development, regenerative processes in the mammalian brain are observed in a region-specific manner.

Neuron loss due to injury or neurodegeneration triggers context-dependent acute or chronic changes that stimulate neural stem/stem-like cells and other injury-responsive cells in the brain. For example, cell death upon injury induces up-regulation of damage-associated molecular patterns, including reactive oxygen species (ROS), which stimulate NSCs to initiate regenerative programmes [6]. However, these alarm signals could alternatively trigger anti-regenerative mechanisms through astrocytes and microglia, leading to neuroinflammation and glial scarring, similar to fibrotic scars in other tissues [7,8].

Most of our knowledge of the cellular and molecular mechanisms of central nervous system regeneration derives from studies on axon regeneration in the spinal cord of zebrafish, axolots, larval *Xenopus* and rodents [9–11]. Axon regeneration refers to the process of restoring neural connectivity. On the other hand, cell regeneration, albeit rare in the adult mammalian brain, is the process of replacing or restoring damaged or missing cells via new cell production. For example, oligodendrocyte progenitors have been extensively studied for their cellular regenerative potential in the mammalian brain [12]. Although scarce, studies exploring regenerative potential during brain development and/or outside the canonical niches provide crucial insights into the regeneration of new cells in the mammalian brain. Here, we summarise the endogenous repair mechanisms to replace cells that occur upon acute cell loss such as stroke, traumatic brain injury or experimental ablation in the developing and adult mammalian brain from a stem cell biology and astroglia perspective. We discuss the cell-intrinsic and -extrinsic changes that occur once development ends and upon ageing and their impact on the NSCs’ regenerative potential. Finally, we conclude by discussing potential therapeutic strategies to facilitate regeneration in the brain.

## Regeneration in the canonical neurogenic niches and cerebral cortex

The developing brain has the potential for regeneration, likely facilitated by the plastic nature of its cells and the permissive microenvironment. The regenerative ability of the embryonic neocortex can be demonstrated indirectly through grafting experiments into the adult injured cortex. In these studies, dissociated embryonic neurons that were transplanted into the cortex integrated into the existing cortical circuits of the adult brain after an injury (e.g. stroke) [13,14]. Interestingly when embryonic tissue was grafted, instead of dissociated neurons, engraftment efficiency increased [15]. Although indirect, these studies highlight the regenerative potential of the embryonic brain and suggest that this is a combination of tissue microenvironment and cell-intrinsic properties.

**Figure 1.**
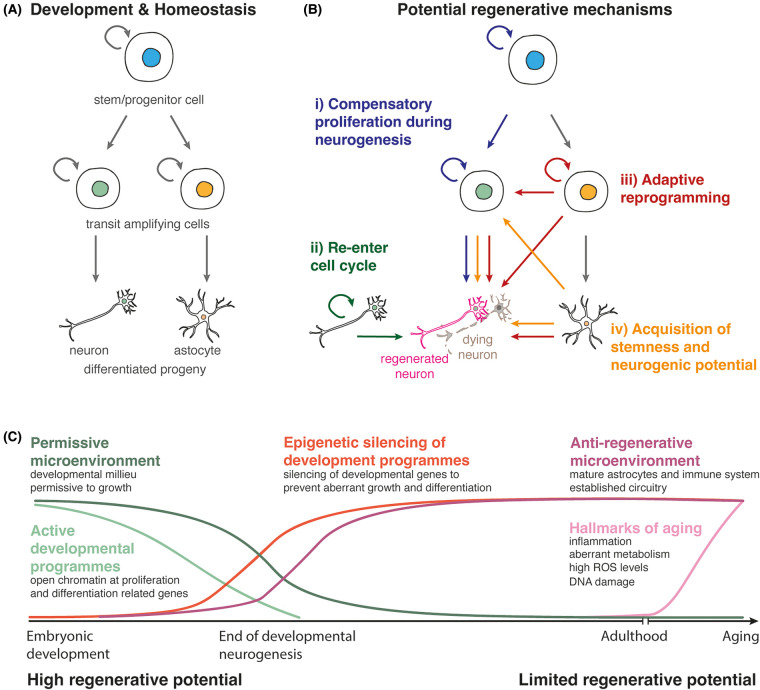
Potential regenerative mechanisms and age-dependent changes in the brain. (**A**) Hypothetical schematic of neural stem cell (NSC) lineages during development and homeostasis (oligodendrocytes were omitted). (**B**) Potential mechanisms of neuron regeneration mediated by (i) compensatory proliferation of NSCs or transit amplifying cells [18–20] (blue), (ii) immature neuron progenitors or neurons re-entering the cell cycle [29] (green), (iii) adaptive reprogramming of NSCs, gliogenic progenitors or astroglia [28,32–35] (red), (iv) astrocytes acquiring stemness or neurogenic potential upon injury [25,26] (yellow). (**C**) Cell-intrinsic and -extrinsic changes that occur during development and ageing in the brain.

In the postnatal brain, canonical neurogenic niches maintain regenerative capacity once development ends and throughout adulthood. One observed regenerative mechanism is the increased compensatory proliferation of endogenous NSCs (Figure 1A,B). In the postnatal brain, the two canonical neurogenic regions: the subventricular zone (SVZ) lining the lateral ventricle and the subgranular zone (SGZ) of the hippocampal dentate gyrus, house NSCs that generate the olfactory bulb and hippocampal neurons, respectively [4,5,16,17]. Studies have reported that, upon injury, undifferentiated neuroblasts from the SVZ proliferate and migrate to the site of injury. In a model of cortical stroke, this migration has been shown to occur along an astrocytic tunnel controlled by several factors including chemoattractants, growth factors (e.g. VEGF) and neurotrophins (e.g. BDNF) that connect the SVZ to the injured non-regenerative cortex [18,19]. Indeed, migration of immature neuroblasts is associated with the expression of Doublecortin-positive (DCX^+^) cells, a marker for immature neurons of the SVZ and SGZ, to the site of injury. However, the origin of DCX^+^ cells in the cortex following injury remains elusive. Evidence of endogenous NSC-mediated regeneration has also been described in the SGZ of the hippocampus where newly generated neurons were observed *in situ* after induced traumatic brain injury [20] and, in an oligodendrocytes cell death model, DCX^+^ proliferating progenitors were associated with the generation of mature oligodendrocytes during hippocampus remyelination [21]. However, the poor survival of the newly generated neurons raises the question of whether such injury responses can be considered as successful regeneration [22,23].

Besides NSCs, many studies highlight the glia, specifically astrocytes, as potential cellular sources for regeneration by taking on stem cell-like capacities upon damage (Figure 1A,B). For example, upon injury to the cerebral cortex, DCX expression could be a result of neuroblast migration to the site of injury [24]. However, other studies suggest that the astrocytes present at the site of injury start expressing DCX [25] and adopt stem-like characteristics. Reactive astrocytes isolated from the acutely injured brain were able to take on NSC characteristics *in vitro* in response to sonic hedgehog (SHH) signalling [26]. Similarly, some specialised astrocytes outside of the cerebral cortex such as Bergmann glia of the cerebellum are transcriptionally similar to the radial glia [27], raising the question of whether they maintain any neurogenic potential. Collectively, these studies provide examples of regeneration via compensatory proliferation within the NSC lineage or acquisition of stemness and neurogenic potential of astroglia around the injury site (Figure 1A,B).

## Regeneration outside the cerebral cortex

Following cell loss, efficient repair has been observed outside of the canonical neurogenic niches (SVZ and SGZ) in an age-dependent manner. The neonatal mouse cerebellum exhibits high regenerative potential upon loss of at least two of its cell types via distinct mechanisms [28–30].

One such mechanism is the re-entry of immature neurons into the cell cycle to replenish their lost neighbours (Figure 1A,B). Purkinje cells are GABAergic neurons that converge all the information that enters the cerebellar cortex to the primary output neurons of the cerebellum, the excitatory cerebellar nuclei. While Purkinje cells are produced between embryonic days (E) 10–13 in mice, they remain immature until completion of postnatal neurogenesis in the cerebellum, two weeks after birth. In one study, ablation of ∼50% of the Purkinje cells at postnatal day (P) 1 resulted in a subpopulation of Purkinje cells, immature Purkinje cell progenitors, re-entering the cell cycle. These cells subsequently replenished their lost neighbours within a day, allowing development to progress normally [29]. Although previously, forcing neurons to proliferate led to cell death [31], this suggests that, within critical developmental windows, injury or cell death could stimulate other immature neurons to re-enter the cell cycle for regeneration.

Another mechanism involves adaptive reprogramming, which comprises the generation of a temporary pool of progenitors/cell states that replenishes the lost cells upon injury or ablation and could exhibit lineage plasticity (Figure 1A,B). During neonatal cerebellar development, molecularly distinct progenitor subpopulations that reside in different regions within the cerebellar cortex produce the requisite cells of the cerebellum. The rhombic lip-derived granule cell progenitors produce the excitatory granule cells, while the ventricular zone-derived *Nestin*-expressing progenitors (NEPs) generate Bergmann glia, astrocytes and inhibitory neurons via molecular distinct subpopulations that reside in different regions within the cerebellar cortex. Upon death of the granule cell progenitors via irradiation or genetic ablation, a subpopulation of the gliogenic *Hopx-*expressing NEPs that normally generate Bergmann glia, regenerate granule cell progenitors via adaptive reprogramming in a multistep response that involves proliferation, fate-switch, migration and differentiation [28,32]. Single-cell transcriptomics on control and injured NEPs, fate mapping experiments and loss of function studies reveal that an *Ascl1^+^* transitory cell state is required for adaptive reprogramming, likely by mediating the gliogenic-to-neurogenic fate switch [32]. While it is still unknown why the loss of different neurons in the cerebellum triggers different regenerative responses, the inherent regenerative capacity of the neonatal cerebellum makes it a powerful model for studying the molecular and cellular mechanisms of regeneration in the brain.

Interestingly, although not spontaneous, the mammalian retina can also show regenerative potential. Manipulation of cell cycle or cellular reprogramming through ASCL1-mediated transcriptional changes and subsequent neuron production has also been observed in the injured mammalian retina. Here, Müller glia act as a source of progenitor cells capable of de-differentiation to a progenitor state, proliferation and neuronal differentiation upon acute retina damage in an age-dependent manner [33–36] (Figure 1A,B). In this instance, Müller glia share a similar transcriptional profile with retinal progenitors, however, key pro-neural genes are not expressed upon injury. As a result, in contrast with the neonatal cerebellum, the retina doesn't undergo spontaneous reprogramming and requires exogenous manipulation of transcription factors such as ASCL1 to initiate their neural differentiation and pro-regenerative program [33,35].

Recently, new putative neurogenic sites have been speculated to exist in the mammalian brain. Although neurogenic potential is low compared with the canonical niches, the hypothalamus, a region important for homeostatic regulation of physiological processes, is an emerging neurogenic region. The tanycytes, radial glia-like cells in the hypothalamus, exhibit stem/progenitor potential both during homeostasis and upon physiological perturbations such as changes in dietary, environmental and hormonal signals [37–39]. However, hypothalamic neurogenesis in response to injury remains to be confirmed but, overall, these observations indicate another cell type with regenerative potential in the brain. In summary, these results demonstrate that different brain regions adopt various regenerative mechanisms to overcome cell loss and highlight the importance of investigating outside the canonical neurogenic niches.

## Age-dependent decline in the regenerative potential of the brain

While distinct context-dependent regenerative responses have been observed in different brain regions, one uniform factor across all these responses is that they are tightly regulated by age. While the developing brain has compensatory mechanisms upon injury or neuron loss, the adult brain has limited regenerative potential outside the neurogenic zones. In addition, adult NSCs become quiescent and exhausted with age, as they exhibit hallmarks of ageing such as accumulated DNA damage and oxidative stress (Figure 1C) [40]. In turn, these collectively dampen the regenerative potential of NSCs. In this section, we will summarise and speculate on the cell-intrinsic and -extrinsic mechanisms of regeneration blockage in the brain and how age influences them. Understanding these molecular and cellular mechanisms is the crucial first step to facilitate repair.

## Cell-intrinsic mechanisms

The state of the epigenome could dramatically influence the proliferation and differentiation potential and hence the repair capacity of the stem/progenitor-like cells after an injury or cell loss. As neural development progresses, NSCs undergo epigenetic changes and switch their fate from neurogenic to gliogenic [41]. The proliferation and differentiation pathways are silenced in the differentiated progeny, potentially to prevent aberrant reprogramming as a protective mechanism against overgrowth or cancers. In addition to the chromatin remodelling that marks the end of differentiation, upon ageing, alterations in the epigenome correlate with an increase in inflammatory pathways [42]. Furthermore, studies comparing the epigenome and transcriptome of adult and aged NSCs in the SVZ show the silencing of genes that are fundamental for stem cell proliferation and differentiation [43–46]. However, it is important to note that these epigenetic features are cell type- and context-dependent and most of these studies are done *in vitro* using primary NSC cultures or they are restricted to neurogenic zones. Therefore, comparative epigenomic analyses in the emerging regenerative sites discussed earlier, such as the cerebellum, as well as *in vivo* functional analyses are required to further elucidate the age-dependent epigenetic mechanisms affecting the regenerative potential of NSC and NSC-like cells. An open question is to what extent the developmental reprogramming of the epigenome during differentiation vs. the effects of ageing on the epigenome influences regeneration.

Among the factors affecting NSC function, and hence regeneration, is cellular metabolism [47,48]. Mitochondria are known to play a pivotal role in energy metabolism and stress response. Importantly, mitochondrial dysfunction is considered another hallmark of ageing [40]. During differentiation, NSCs undergo a metabolic switch from glycogenic to oxidative phosphorylation and the mitochondria morphologically change to accompany this transition [49,50]. Products of mitochondrial metabolism such as endogenous ROS are shown to regulate NSC self-renewal and differentiation in a transgenic mouse model that reduces ROS levels [6]. However, in the aged brain, accumulation of mitochondrial DNA mutations increases ROS levels, impairs the oxidative phosphorylation machinery and causes DNA damage. These alterations significantly impair neurogenesis and support the change in NSC lineage decisions from a neuronal fate to an astroglia one [51–54]. Collectively, metabolic changes in NSCs during differentiation or ageing could indirectly affect their regenerative potential. Interestingly, although ROS is a damage-associated molecular pattern and is increased in the microenvironment upon cell death, whether the endogenous ROS levels of NSCs or other injury-responsive cells factor into the regenerative response remains to be studied.

## Cell-extrinsic mechanisms

Inflammation and scar formation are known to be inhibitory to regeneration in many complex tissues. Upon injury and acute cell loss, brain parenchyma also initiates inflammatory responses. In addition, reactive astrocytes change morphology and transcriptional profile, and importantly, activate proliferation programmes in response to damage-associated molecular patterns, leading to gliosis. In most cases, inflammation and reactive gliosis are thought to be inhibitory to regeneration, while context-dependent nuances may exist [55,56].

Microglia, the resident macrophages of the brain, are derived from the embryonic yolk sac [57]. Microglia are commonly classified into a pro- or anti-inflammatory phenotype. However, recent studies have shown that microglia have vast molecular and cellular heterogeneity across different brain regions and, importantly, between the developing and the adult brain [58]. Furthermore, forebrain organoid models that include microglia showed modulation of NSC behaviour, highlighting the cross-talk between microglia and NSCs during development [59]. Finally, IFNy and cGAS-STING DNA sensing pathways have been shown to drive neuroinflammation degeneration and ageing, further confirming the importance of the immune system on nervous system pathophysiology [60]. However, the functional relevance of microglial heterogeneity and the cross-talk between microglia and NSC/NSC-like cells during regeneration and repair remain to be understood. Upon injury to the brain, an inflammatory response is initiated by the microglia in a context-dependent manner which, in turn, secrete pro-inflammatory molecules such as cytokines and chemokines to the site of injury [61–63]. While microglia are required for regeneration in the zebrafish spinal cord [64], inflammation could lead to reactive gliosis and a glial scar formation in the adult brain, which are traditionally thought to be inhibitory to regeneration.

Circulating immune cells are also known to have an inflammatory role upon injury with long-term effects. At homeostasis, the blood-brain barrier regulates infiltration into the brain. However, upon injury or ageing, the barrier could break down, allowing the infiltration of immune cells such as T cells into the brain parenchyma leading to inflammation [65,66]. Furthermore, meningeal immune cells also release cytokines to stimulate cells in the brain [67]. Studies on the SVZ NSCs showed antiproliferative effects of IFNy, suggesting implications for NSC function during regeneration [66]. These findings are strikingly different to what happens in the neonatal brain where the presence of an immature immune system promotes hippocampal neurogenesis and spatial learning processes, following CD4^+^ T cell infiltration [68,69].

The astrocytes represent the most abundant glial cell population in the brain. Under physiological conditions, they are involved in synapse formation, neuronal protection and blood brain barrier support [70,71]. Injury or damage in the brain leads to immune cell infiltration and an increase in oxidative stress, which in turn triggers a signalling cascade that changes the molecular and morphological profile of the local astrocytes, leading to reactive gliosis and glial scars [72,73]. Whilst being anti-regenerative, the scar represents a defensive mechanism against the expansion of the damage to the surrounding healthy tissue [74].

One of the obvious differences between the developing and adult brains is the composition and the molecular identity of microglia and astrocytes. One hypothesis is that the changes in numbers, morphology, functional and molecular identity could be an underlying reason behind the regenerative developing and non-regenerative adult brain. In addition to the immune and glial microenvironment, brain parenchyma undergoes many other changes after development. For example, changes in extracellular matrix composition and increased stiffness were linked to reduced regeneration by oligodendrocyte progenitor cells [75], suggesting other stem populations could also be affected by such changes. Furthermore, although examples of the integration of new neurons into the brain exist [76,77], once the circuitry is established and mature, there could be inhibitory cues that prevent the integration of new neurons into the circuitry.

In summary, a combination of cell-intrinsic and -extrinsic changes influence the repair capacity of the brain (Figure 1C). Importantly, the age-dependent decline in the regenerative potential is multifactorial and requires innovative combinatorial approaches to overcome various effectors. Heterochronic transplantation and parasymbiosis [78] experiments could provide further insights into the contribution of different cell-intrinsic and extrinsic factors.

## Potential therapeutic approaches to facilitate brain regeneration

There are no regenerative therapeutic approaches after an injury or acute cell loss in the brain. Current treatments mainly include therapies that target the after-effects of the injury and therefore do not replace/regenerate the lost neurons [79].

While cell replacement therapies are promising for some degenerative diseases such as Parkinson's Disease [80], transplantation approaches may not always be feasible when the injury spans multiple cell types and large areas. Therefore, direct reprogramming *in vivo* is emerging as a promising therapeutic approach to stimulate new neuron production. Astrocytes represent the ideal target for *in vivo* reprogramming, given their potential stem-like properties upon injury [26]. Furthermore, since they drive reactive gliosis upon brain damage, altering astrocyte function and lineage propensity could facilitate new neuron production and alleviate the anti-regenerative microenvironment. This has been achieved through overexpression of proneural transcription factors such as *NeuroD1*, *Ascl1* and *neurogenin2* which are necessary and sufficient to trigger a neuronal fate in experimental models [81–85]. Overexpression of these transcription factors changes the astrocytic chromatin landscape by opening the silenced proneural genetic loci and therefore activates neuronal genetic programs. For example, ASCL1 induces a rapid global change in the transcriptional program of reprogrammed astrocytes. This is achieved by directly binding to its target genes *Klf10*, *Myt1*, *Neurod4* and *Chd7*, required for the efficient conversion of astrocytes into neurons [83]. A crucial consideration following direct reprogramming is to assess whether the correct neural identity is achieved and the safety of the gene delivery approaches. Importantly, while direct reprogramming could short-circuit neuron production, it does not overcome the potential anti-regenerative effects of tissue microenvironment. For example, in one study, microglia in the microenvironment have been shown to inhibit the *Ascl1*-induced retinal regeneration in adult mice [86].

Alternatively, stimulation of endogenous stem/progenitor cells through transient manipulations represents another potential therapeutic approach to overcome the cell-intrinsic and extrinsic regeneration blockages. However, we hypothesise that such approaches should be combinatorial, targeted and transient. After traumatic brain injury, injections of growth factors such as EGF and FGF2 in the ventricle of adult rodents increased the proliferation of NSCs, supporting neuron formation and cognitive recovery [87,88]. Whether providing developmental signals and mitogens could be sufficient to overcome the epigenetic silencing of neural differentiation genes and reprogramme the cells to make neurons remains to be determined. Collectively, these findings highlight the need for combinatorial approaches that tackle cell-intrinsic and -extrinsic factors. Therefore, understanding the molecular and cellular mechanisms regulating injury responses and the effects of age is crucial to fully harnessing the potential of endogenous stem cells.

## Conclusions and outlook

Although the adult brain is inefficient at making new neurons after cell loss due to an injury or degeneration, the developing brain and active neurogenic niches exhibit regenerative potential. Regenerative mechanisms could involve compensatory proliferation of NSCs or adaptive reprogramming of injury-responsive progenitors or astroglia. Nevertheless, these processes are tightly regulated with age, with the majority not observed after development. The decline in regenerative potential is likely an evolutionary trade-off to achieve complex circuitry and higher-order function and to prevent aberrant growth and tumorigenesis.

Our current understanding highlights the need for combinatorial approaches that modulate the adverse effects of the injury microenvironment and reverse the cell-intrinsic changes that accumulate over time. Investigating the regenerative processes within the neurogenic niches and elsewhere in the brain, and, finally, contrasting mechanisms observed in highly regenerative organisms, could provide invaluable insights into how to stimulate neuron regeneration following injury.

## Perspectives

- The adult mammalian brain exhibits limited regeneration outside the neurogenic niches.
- Developing brain exhibits repair capacity highlighting the age-dependent decline in the regenerative potential in the brain.
- A combination of cell-intrinsic and -extrinsic factors regulate the regeneration in the brain and understanding these factors is the crucial first step for the design of potential future therapies to facilitate regeneration.

## Open Access

Open access for this article was enabled by the participation of University of Cambridge in an all-inclusive *Read & Publish* agreement with Portland Press and the Biochemical Society under a transformative agreement with JISC.

## Abbreviations

NEP: *Nestin*-expressing progenitor
NSC: neural stem cell
ROS: reactive oxygen species
SGZ: subgranular zone
SHH: sonic hedgehog
SVZ: subventricular zone
